# A Mass in the Thorax: A Rare Presentation of Gastrointestinal Stromal Tumor

**DOI:** 10.7759/cureus.22601

**Published:** 2022-02-25

**Authors:** Kristen L Farraj, Aboud Kaliounji, Jiten Desai, Kevin Yeroushalmi, Nausheer Khan

**Affiliations:** 1 Internal Medicine, Nassau University Medical Center, East Meadow, USA; 2 Internal Medicine, St. George's University School of Medicine, New York, USA; 3 Gastroenterology, Nassau University Medical Center, East Meadow , USA; 4 Gastroenterology, Nassau University Medical Center, East Meadow, USA

**Keywords:** extraintestinal gastrointestinal stromal tumors, clinical pathology, immunohistochemistry staining, gastrointestinal stromal tumours, mesenchymal cells

## Abstract

Gastrointestinal stromal tumors (GIST) can occur anywhere in the gastrointestinal tract, with the primary site being the stomach. GISTs are often discovered incidentally on imaging due to the non-specific symptoms they present at the presentation. They can be distinguished from other mesenchymal tumors by immunohistochemistry staining. These tumors can range from benign to highly malignant, with surgical resection as the main treatment modality. Here, we present a case of a large GIST found not in the stomach, but in the mediastinum, incidentally on imaging, in a middle-aged male.

## Introduction

Gastrointestinal stromal tumors (GIST) are the most common mesenchymal tumors that arise from mesenchymal cells in the gastrointestinal (GI) tract [1,2]. Although sporadic and rare, they can be found anywhere along the gastrointestinal tract, but are mainly located in the small bowel and stomach [3]. Today, GIST includes leiomyosarcomas, leiomyoblastomas, schwannomas, and leiomyomas, which were previously considered GI soft tissue neoplasms [1].

Around 20% of the cases are found incidentally on imaging or during procedures for other reasons, such as surgery or endoscopy [3]. Patients with GIST commonly present with abdominal pain and GI bleeding, though the majority remain asymptomatic [3,4]. Overexpression of the tyrosine kinase receptor KIT is characteristic of these tumors [5]. Imaging such as computed tomography (CT), magnetic resonance imaging (MRI), and endoscopy with or without ultrasound are useful for diagnosis. Biopsy by fine-needle aspiration and immunohistochemistry confirms the diagnosis but is not always indicated due to the high risk of hemorrhage and tumor dissemination [1]. Histological findings include cellular proliferation composed of either epithelioid or spindle cells [4]. 

While GIST can be benign or malignant, surgical resection is the ultimate treatment along with pharmacological treatment with the tyrosine kinase inhibitor Imatinib in patients with a malignant tumor [2]. Prognosis, however, includes mainly tumor size and mitotic activity, as well as primary location and other minor factors [1]. In this clinical case, we describe the presentation of a middle-aged Hispanic male who was found to have a benign GIST.

## Case presentation

**Figure 1:**
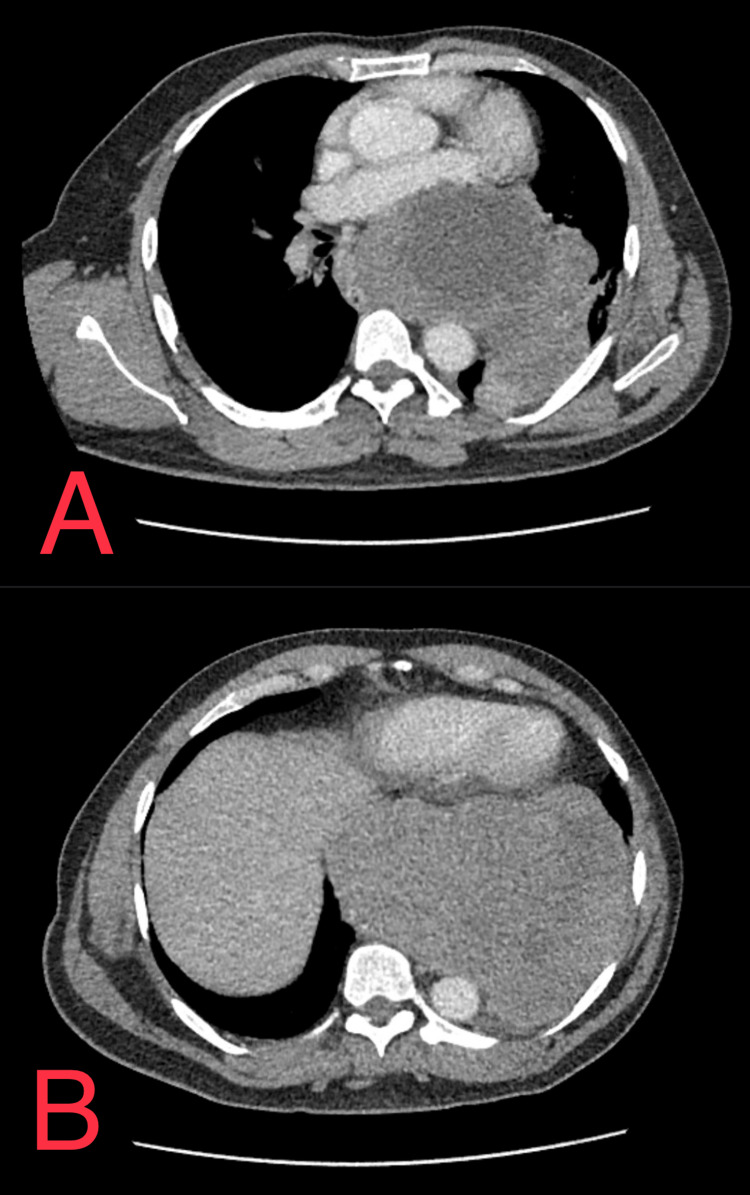
(A) The mass extending from the esophagus into the lower mediastinum compressing the heart and displacing the aorta completely to the left. (B) The large lobulated heterogeneous mass measuring 24 cm at its largest.

A 45-year-old Hispanic male with a past medical history of hypertension presented to the emergency department with intermittent, non-exertional, non-radiating substernal/epigastric chest pain for a six-month duration. The pain is accompanied by dysphagia when eating solids and liquids. A CT scan of the chest with intravenous (IV) contrast was performed, which was positive for a very large lobulated left paraesophageal lower mediastinal mass extending through the esophageal hiatus into the left upper abdomen along the greater curvature of the stomach. The mass contains multiple hypodense areas consistent with necrosis, as well as a few scattered dystrophic calcifications. Also, the mass displaces and narrows the middle and lower esophagus, the gastric fundus, and upper gastric body without significant dilatation of the proximal esophagus above the mass, and displaces the heart anteriorly with extrinsic compression of the left atrium, as seen in Figure 1.

**Figure 2:**
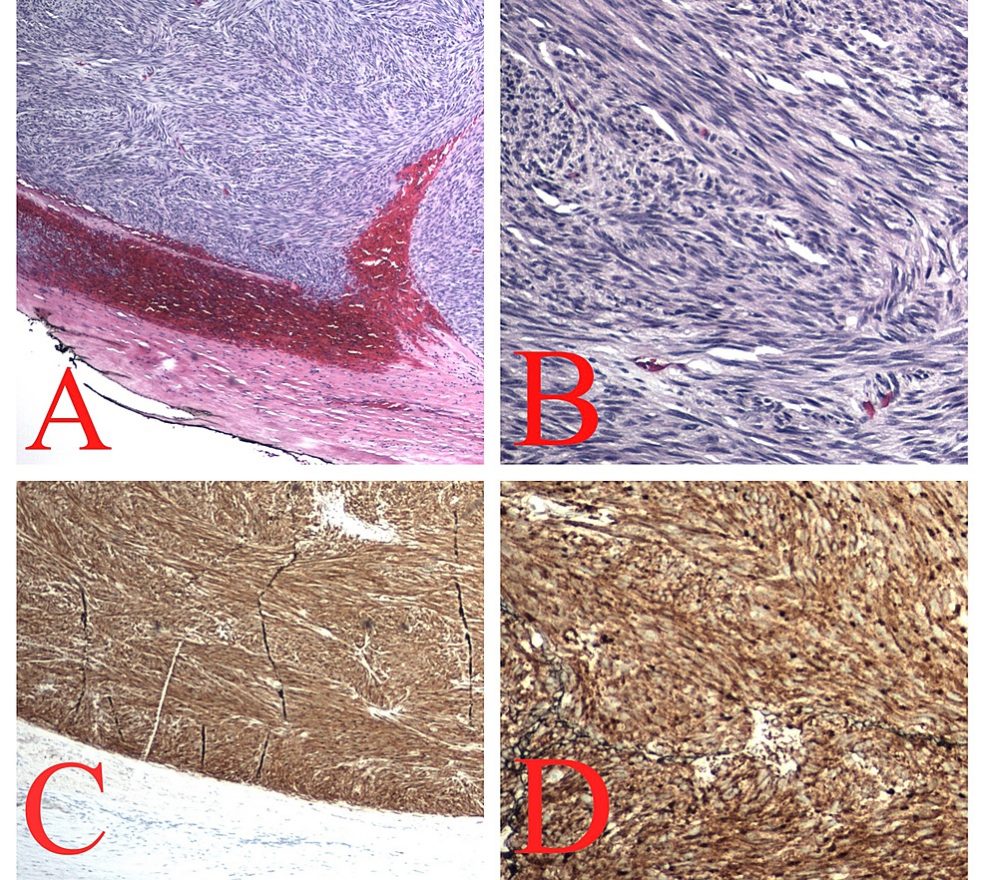
(A) Demonstrates spindle cell tumor adjacent to residual smooth muscle fibers at 40× power field; (B) demonstrates the neoplastic spindle cells at 200× power field; (C) demonstrates the positivity of the neoplastic spindle cells for CD117 on immunohistochemistry; (D) demonstrates the positivity of the neoplastic spindle cells for DOG-1 on immunohistochemistry.

The gastroenterology team was consulted, and an esophagoduodenoscopy (EGD) was performed to further evaluate the mass. The mass was not well visualized on EGD, but extrinsic compression of the esophagus was seen, and biopsies were positive for Helicobacter pylori infection. Triple therapy, which included amoxicillin, clarithromycin, and pantoprazole, was initiated during a hospital stay. Interventional radiology (IR) was consulted in order to biopsy the mass. The mediastinal and abdominal masses were biopsied and the initial pathology was positive for a malignant spindle cell. Immunohistochemistry staining was positive for CD117, CD34, and DOG-1, as seen in Figure 2.

This supported the morphologic diagnosis of spindle cell neoplasm and GIST. At that time, the oncology team had discussed neoadjuvant therapy with imatinib prior to surgical removal with the patient, but he wished to proceed with surgery and initiate imatinib after the operation. The surgical team was then consulted for excision of the mass, and an exploratory laparotomy was scheduled. The stomach was inspected and the mass was noted to be originating from the gastroesophageal junction (GEJ). The mass was noted to be coursing through the hiatus, very adherent at the GEJ, and involving a large portion of the medial diaphragm extending through the chest superiorly, just abutting the aorta at the arch, and was adherent to the lung parenchyma laterally. He subsequently underwent a left thoracotomy, resection of the thoracoabdominal mass, and esophageal and stomach resection with gastroesophageal anastomosis. There were no intraoperative complications; the estimated intraoperative blood loss was 30 cc; and the total operative time was five and a half hours. No metastatic disease was noted and the margins were negative for any gross or microscopic tumor cells. The patient remained in the surgical intensive care unit for six days post-op as the patient was unable to be extubated until post-op day 5. One day post-extubation, the patient was transferred to the medical floors for further management. Neoadjuvant therapy was still withheld at this time as the patient was not medically optimized. Unfortunately, on the patient's second day on the medical floors, a rapid response was called for as the patient was found to be acutely distressed, diaphoretic, tachycardic with a heart rate of 134 beats per minute, and hypotensive with a mean arterial pressure of 47. A femoral central venous catheter was placed for vasopressor support, and the patient was re-intubated in the room. He was then transferred back to the intensive care unit. A code blue was called shortly after the transfer for pulselessness and unresponsiveness. Asystole was noted on an electrocardiogram. Advanced cardiovascular life support (ACLS) was initiated with multiple rounds of cardiopulmonary resuscitation, but the family ultimately decided to make the patient "Do Not Resuscitate and Do Not Intubate" (DNR/DNI), and he was pronounced deceased. At the time of death, the probable cause was deemed to be a massive pulmonary embolism.

## Discussion

Prior to the identification of GIST tumors, mesenchymal tumors were thought to be smooth muscle tumors, such as leiomyomas and leiomyosarcomas [6]. Mazur and Clark first introduced GIST in 1983 and defined it as a non-smooth muscle mesenchymal tumor characterized by the overexpression of c-KIT or CD117 and CD34, as seen in our patient’s immunohistochemistry panel [7]. The identification of GIST tumors was dependent on technological advances, specifically in immunohistochemistry. Gastrointestinal bleeding and pain are the most common symptoms on presentation, but an incidental mass on CT scan is what leads the physician to biopsy and eventual diagnosis. Similarly, in our case, his main complaint was constant abdominal and chest pain. Furthermore, while our patient did not have any signs of a GI bleed, more than 50% of GIST patients usually present with GI bleeds that can be massive, requiring urgent treatment, or chronic with anemia [5].

In their systematic literature search of 19 population-based epidemiological reports, Soreide et al. reported that the incidence of GIST in most studies was 10-15 per million per year [8]. Norway, Taiwan, Hong Kong, and Shanghai areas reported the highest incidence, recording around 19-22 per million per year. On the other hand, North America reported a very low incidence of 4.3 to 6.8 per million per year. They also added that it is predominantly found in people who are in their 60s, with an age at diagnosis ranging from 10 to 100 years old and presents equally in males and females.

In 2002, Fletcher et al. created a classification system for malignancy grading. He divided it into four categories: very low, low, intermediate, and high-grade malignancy [9]. Any GIST that is greater than 5 cm or any size but has more than 10 mitoses per 50 fields is considered high grade and has a higher chance of recurrence. In contrast, a very low grade is any GIST less than 2 cm with fewer than five mitoses per 50 fields. In our case, it met high-grade malignancy due to its size of 24 cm, but it only had five mitoses per 50 fields. 

To date, there is only one other case discussing a GIST found outside the parameters of the gastrointestinal tract. Zongo et al. discussed a retroperitoneal GIST found incidentally on CT imaging due to the patient's complaining of lumbar swelling and mild discomfort with movement [10]. Similarly to our case, the patient was a middle-aged male and the CT scan was the key to discovering the tumor. The mass in their case was also larger than 10 cm and was surgically resected.

Surgery is the main form of treatment, with imatinib added as a combination therapy in metastatic or unresectable cases of GIST. After surgical treatment, benign GISTs have a 90% five-year survival rate, while malignant or recurrent GISTs have an average survival of one year [1]. However, treatment for mediastinal GISTs has not been standardized due to its rarity, and in our case, the patient expired within two weeks of resection. In contrast, the patient with the retroperitoneal GIST discussed above survived the surgical resection and displayed no signs of recurrence. This poses a significant question about the treatment plan for extraintestinal GISTs and warrants further investigation and studies.

## Conclusions

Despite GISTs being considered a rare gastrointestinal tumor, the prevalence has been increasing due to advances in diagnostic methods. Furthermore, the tumor may extend outside the gastrointestinal tract, as in our patient above. With abdominal pain and GI bleed being the main presentation, it is important for physicians to keep GIST on their differential as early diagnosis can allow for timely management.
